# Forage quality of consecutive years interact to affect body condition, reproductive rate and rut phenology in Iberian red deer

**DOI:** 10.1371/journal.pone.0278367

**Published:** 2022-12-01

**Authors:** Marina F. Millán, Juan Carranza, José M. Seoane, Javier Pérez-González

**Affiliations:** 1 Wildlife Research Unit (UIRCP), Universidad de Córdoba, Córdoba, Spain; 2 Biology and Ethology Unit, Universidad de Extremadura, Cáceres, Spain; Universita degli Studi di Sassari, ITALY

## Abstract

Body condition for reproduction in capital breeders such as the red deer (*Cervus elaphus*) is mostly determined by their stored energy reserves. Thus, environmental conditions and resource availability may affect reproductive performance and breeding success. In warm Mediterranean regions, current climate change is driving to a hotter and drier scenario that is expected to affect the biology and dynamics of many populations. We examined the impact of these local climate variations on red deer body condition and the relationship with female reproductive phenology and breeding success. We used satellite information of landscape vegetation along with a 22-year data series of direct field behavioural observations during the rutting season in Doñana National Park (SW Spain). We analyzed faecal nitrogen content (FN) from faeces collected during the rut. We found that poor vegetation availability in drier years was related to worse body condition of deer (measured by FN) and a delay in the rutting season, which associated with lower reproductive rates (measured by the proportion of females with calves observed the next year). We also evidenced an interesting interaction between environmental conditions in consecutive years on the timing of breeding season, with timing of breeding being more delayed when previous year resource availability was high and many females bred, and the consecutive one was poor, so females hardly recovered condition and the rut occurred later. These findings highlight the carry-over effect of reproduction in capital breeders and the potential impact of climate-change conditions on red deer breeding.

## Introduction

Animal body condition depends on the balance between energy intake obtained by food and the energetic cost of biological processes, such as growth, survival, or reproduction [1]. Seasonal reproduction is affected by physical condition, and is therefore strongly influenced by environmental factors [2].

Current food intake and stored reserves are the two main sources of nutrients and energy that determine the reproductive ability of seasonal breeders [3, 4].

Most polygynous ungulates, such as red deer (*Cervus elaphus*), are closer to the capital end in the continuum between capital-income breeding strategies [2, 5], since they invest a great amount of their accumulated body reserves in reproduction, as opposed to income breeders that rely more on current energy intake, like the roe deer (*Capreolus capreolus*) [5, 6].

Body condition in many ungulates depends on environmental factors that promote food availability and on population density, since high population densities may significantly constrain intake due to intraspecific competition [1, 7–9].

Under harsh climates, female ungulates face seasons of limited resource availability, hindering energy intake for body demands [10, 11]. Body condition noticeably decreases in females during gestation and lactation due to the strong energy investment required, and in males during the rutting season due to the mating effort [5, 12–14]. In female red deer, ovulation and fertility has been related to body mass and fat reserves [13, 15–17]. Thus, when females do not get optimal nutritional status for reproduction, ovulation can delay or even fail [16, 18]. Therefore, environmental changes due to climate change could delay or decrease reproduction rates in the population through changes in resource availability affecting energy balance [19, 20].

For females, minimal body reserves and condition are reached after gestation and lactation, so the recovery of deer body condition strongly depends on spring weather, since it determines the forage onset and availability after the breeding season [21–23]. Moreover, some studies have shown that the conditions of the previous season have an important carry-over effect on capital breeding [2, 24–26]. Reproductive effort affects body reserves, and hence, the ability to reproduce in the next season [27–29]. In red deer, adverse environmental conditions delayed the first year of oestrus or even produced oestrus only in alternate years [29]. Gestation and lactation costs reduced female body condition for next breeding season and.females that did not reproduce were in better body condition at the next mating season [13, 29].

Thus, seasonal breeding ungulates must synchronize their reproductive cycles with the time of green-up so that lactation can occur at the time of resource peak [30]. If females delay oestrus, they might not match their parturition date to the optimal timing of resource availability, affecting their offsprings’ fitness [31]. Indeed, calf weight at birth is related to mother’s weight during pregnancy and lactation, and to calf future weight, which will determine calf survival probability and reproductive success [32].

Therefore, reproduction, fecundity and energy allocation strategies, are closely related to and ultimately regulated by environmental conditions. Hence, current climate change may be altering the reproduction and breeding cycle of red deer, especially in those environments becoming more arid such as those in southern Europe. Environmental changes due to climate change may also affect seasonal reproduction and fitness in red deer by producing a mismatch between actual and optimal calving dates. Several studies have found an advance in parturition and oestrus date in many ungulates in northern Europe in response to an earlier spring, which associated with a longer period of high-quality resource availability [13, 33, 34]. Although little is known about how climate affects reproduction timing of red deer in Mediterranean regions, later conception dates after drier and hotter springs have been related to lower plant productivity [16].

Here, we present a 22-year study that integrates the effect of food availability on the reproduction rates and timing of red deer in Doñana Biological Reserve through the relationship with body condition. In Doñana National Park, in the southwest of Spain, climate is increasingly arid, and the vegetation has changed to plant species better adapted to this type of climate [35–38]. Global change and human pressure in nearby areas, especially with regard to the use of ground water, are increasing the drought events in the area [39–41]. Therefore, we expected that drought periods may affect food availability for red deer, and hence the body condition they can get. In dry years with less plant productivity, the nutritional status of deer may be deficient and ovulation may be delayed or suppressed, so that reproductive rates and female reproductive fitness may decrease.

To test this, we studied the relationship between the NDVI (Normalized Difference Vegetation Index) and the FN (faecal nitrogen content) from feces collected in Doñana during four consecutive years, and between NDVI and rut peak date along a 22-year period, to assess the effects of resource abundance on deer body condition and reproductive phenology respectively. Then, we analyzed the effect of these factors (NDVI, FN and rut peak) on the percentage of offspring per female in the next year, from data obtained through daily observations of the red deer in Doñana, to study how these variables can affect the reproductive rate of the population. Also, we studied the potential carry-over effects that plant availability in a year can exert on the phenology of the following year’s rutting season.

## Material and methods

### Study area

This study was carried out in Doñana Biological Reserve in Southwestern Spain, within the Doñana National Park (Southwestern of the Iberian Peninsula, ca 37°10’N, 6°23’W). Doñana is located in the right bank of the Guadalquivir River and it stands out for the diversity of biotopes. The climate in Doñana is Mediterranean, and the rainy seasons are spring and autumn, so the amount of rainfall in those seasons mostly determines vegetation growth and availability [42]. This area is characterized by hot and dry summers, and the rut occurs in September, just after the period of highest resource scarcity. Our study area was comprised of a western shrub zone and an eastern marsh zone, separated by an ecotone of open meadows with some remaining pasture patches where deer typically aggregated during the mating season [43], and where we located our four study points, each one comprising an area of 70 ha, as done in a previous study [42].

### Rut data and FN measurement

We measured daily population density and roaring rate during the rut along a period of 22 years (1995–2017, except 2007) in four observation sites. We used the mean number of females and calves, and adult, subadult and young males of each observation point as population density estimation of each year. The calves/hind ratio was used as an estimation of reproductive rate, which is in fact a measure of calves that survived close to the independence [44–46]. We quantified the intensity of the rut by counting the number of roars per minute. This measure has been previously used as an indicator of the rut phase [47]. The day with the highest roaring rate was considered the rut peak day, and we used it as an approximation of the main conception date [48].

The faecal nitrogen content (FN) is a good indicator and non-invasive method for assessing nutritional status and diet quality of red deer [49–51]. To evaluate deer nutritional status, we quantified total nitrogen content (FN) from 60 samples of faeces randomly collected in the four study points during the rut in four consecutive years (2011–2014; 15 per year). We tried to avoid repeated samples from the same individuals by collecting fresh samples separated in space. To check whether we got any pseudo-replicated samples, we genotyped faecal DNA at 15 microsatellite loci for the samples from 2011 and 2012. No case of individual repetition was found for those 30 samples (not shown), so we assumed that our sampling procedure was good enough to avoid pseudo-replication and did not genotyped the next-years samples. Total FN content was measured using the EUROVECTOR EA-3010 elementary analyser, which determines the quantitative nitrogen content of the samples.

This work did not have an implication in animal welfare since the data has been obtained through remote observations of the animals and non-invasive sample collection, without any interference or contact with them.

### NDVI

The Normalized Difference Vegetation Index (NDVI) has been previously used in several studies on global change and forage availability for large herbivores in open landscapes [52–55]. We used the NDVI as a measure of resource availability for deer in the area. We calculated the average NDVI value in late spring and summer (May-August), just at the end of vegetation growth and before the rutting season in September. This period proved to be the one with strongest effect on the next rut peak for red deer in Doñana [42]. The NDVI was calculated in QGIS for each year from 450 coordinates distributed throughout the study area to obtain a consistent unique value, as previously used in another study [42, 56]. We averaged monthly NDVI values between May to August obtained from Landsat images courtesy of the U.S. Geological Survey.

We discarded images with high cloudiness, and we applied a previous atmospheric correction. We used the SCP complement (Semi-Automatic Classification Plugin) [57] to apply the DOS atmospheric correction [58], which is one of the most commonly used methods of atmospheric correction.

In order to include NDVI data of all our study period, we used Landsat 7 images from 2000 to 2017, and Landsat 4–5 images from 1995 to 1999, because of the non-availability of images from the same satellite for the whole period.

### Statistical analysis

We elaborated two different datasets, one containing the FN content data (N = 60 samples, 2011–2014) together with data of the mean NDVI, population density, and calves per hind ratio in the rut and in the next rutting season; and the other with data of the whole study period about NDVI and NDVI of the previous year, rut peak date, calves per hind ratios and population density, of each observation point (N = 88, i.e. 22 years x 4 observation points).

First, we explored the effect of the NDVI on the FN content to assess the effect of resource availability on deer body condition. For this, we used the first dataset to build a linear model (model 1) for the dependent variable FN content and the covariates NDVI, population density and current calves/hind ratio (to account for the effort of reproduction of last season). To evaluate the effect of the resource abundance of consecutive years on the rut date, we built a GLM fitted to a gamma distribution for the response variable rut peak date and the fixed factors NDVI, NDVI of the previous year, the interaction between them, and population density, using the second dataset (model 2). Observation point was added as random factor in both models but we removed it later because of the absence of variance due to this factor.

Then, we built three models for the response variable calves/hind ratio and three fixed factors to study their impact on deer reproductive rate: model 3.a) a LM with NDVI, calves/hind ratio of the previous year and population density; 3.b) a GLMM fitted to a gamma distribution with FN content, calves/hind ratio of the previous year and population density; and 3.c) a LM with rut peak date, calves/hind ratio of the previous year and population density. We did not include the three variables in a single model because FN data proceeded from the four-year (2011–2014) dataset, and because the effects of the NDVI and rut peak date were related so the effects were lost when adding them together in the model. We added the calves/hind ratio of the previous year in the models to control for the female reproductive effort in the previous year. We also included population density as explanatory variable in the models since the number of deer in the area may affect rut date and body condition [29, 59–61], as females in high-density areas may have poorer body condition than those in low-density areas, so they may be more strongly affected when resources are scarce [62].

We added again the random factor observation point but removed it from the models 3.a) and 3.c) because of the absence of variation explained by this factor. We also explored the effects of all double interactions between the covariates in the models and then removed them because none of them was significant.Finally, we visually represented the single effect of the three study variables of the analysis using linear regressions with the predicted values of the response variable calves/hind ratio and also showing the raw data in each plot.

All the analyses were conducted in the R version 3.5.2 [63] using lme4 [64] and mgcv [65] packages.

## Results

**Fig 1:**
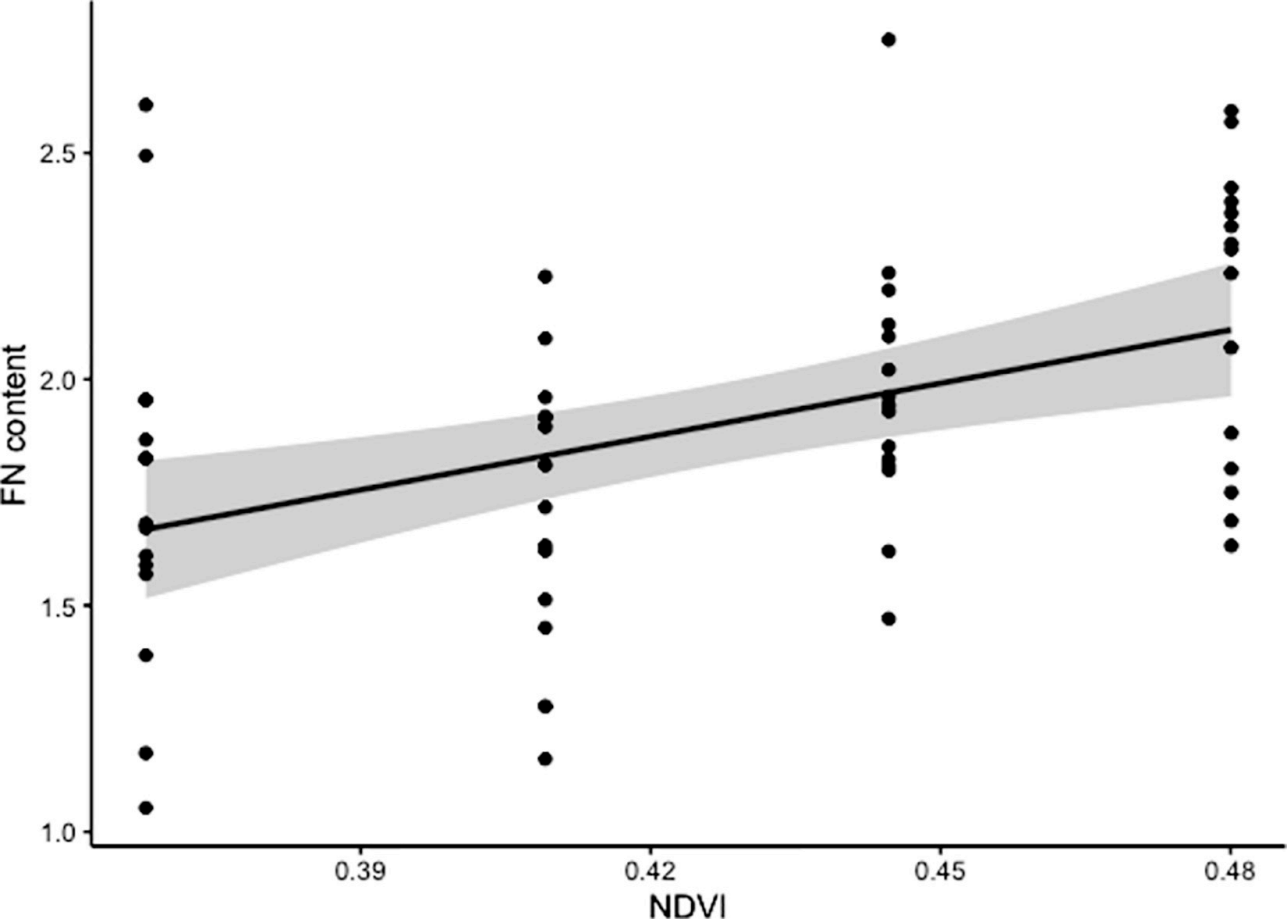
Relationship between mean NDVI in spring-summer and FN content during the rut, from model 1. Line represents model prediction when all other variables are set to their mean values. Points are raw observed data. Shaded areas denote 95% confidence intervals.

NDVI in late spring and summer (May-August), before the starting of the rutting season, positively affected the FN content during the rut (September; Table 1, Fig 1), indicating better body condition with higher vegetation availability. Population density and calves/hind ratio did not show a significant effect in the model to explain FN content (Table 1).

**Table 1:** Results of model 1 for the response variable FN content and the covariates NDVI in spring-summer, population density and calves/hind ratio.

	Estimate	SE	t value	P
Intercept	1.895	0.045	42.217	< 0.001***
NDVI	0.190	0.050	3.775	< 0.001***
Population density	0.048	0.050	0.963	0.340
c/h ratio	-0.030	0.046	-0.665	0.509

**Fig 2:**
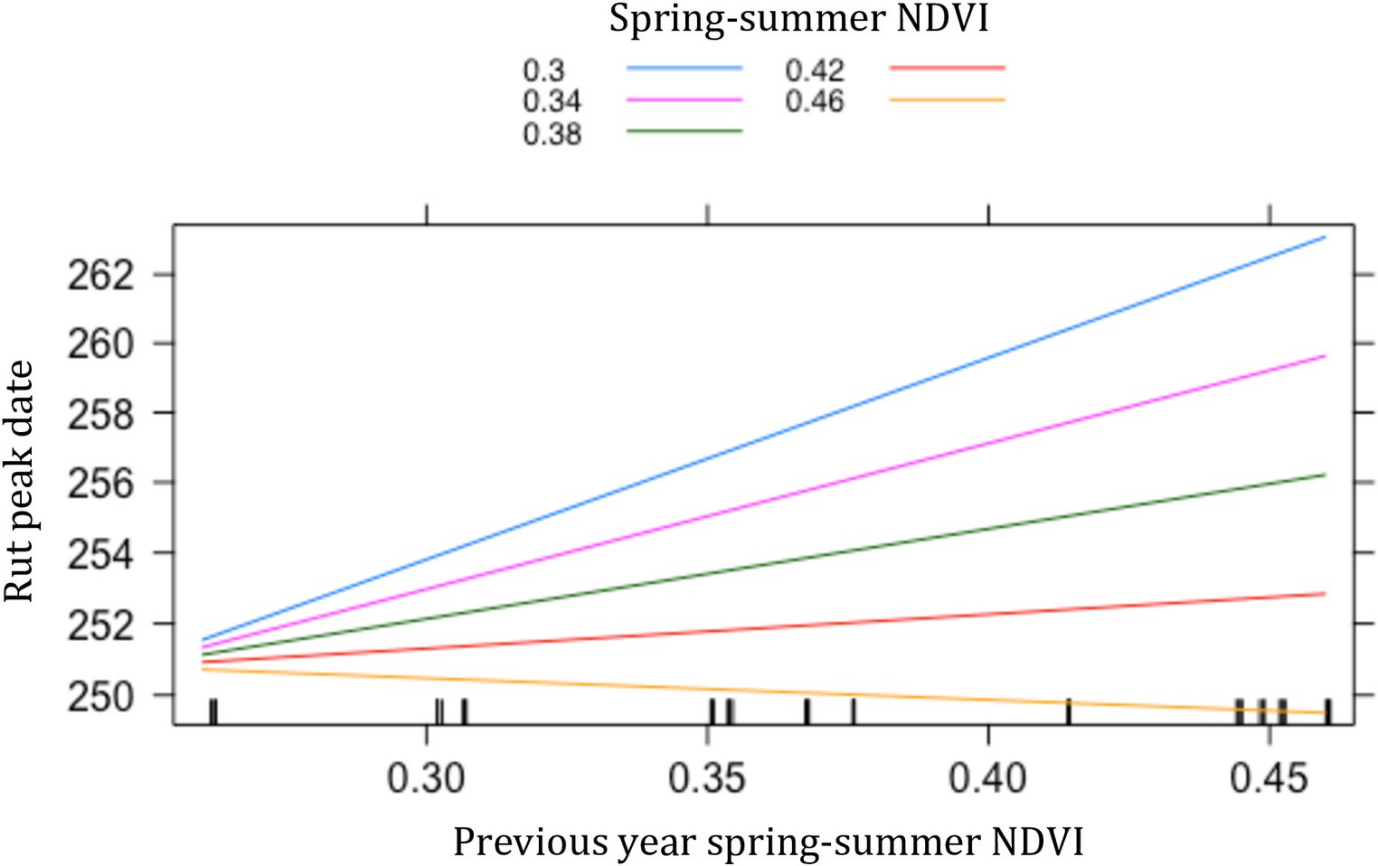
Effect of the interaction between the spring-summer NDVI and the NDVI of the previous-year spring-summer on the rut peak date, from model 2. Figure shows predicted relationships between previous-year NDVI and the rut peak date for different values of spring-summer NDVI in the current year.

The effect of the spring-summer NDVI on the rut peak date interacted with the spring-summer NDVI of the precedent year to determine the peak of the rut (Table 2). When the spring-summer NDVI was low, the rut tended to delay, and the delay was more pronounced when NDVI in the previous-year spring-summer was high rather than low. However, when the current year spring-summer NDVI was high, the rut occurred always early, regardless of the NDVI of the previous year (Fig 2).

**Table 2:** Results of model 2 for the response variable rut peak date and the covariates NDVI in spring-summer just before the rut and NDVI in spring-summer of the precedent year (t-1).

	Estimate	SE	t value	P
Intercept	5.377	0.002	2363.421	< 0.001***
NDVI	-0.014	0.003	-4.696	< 0.001***
NDVI(t-1)	0.006	0.002	2.517	0.016*
Population density	-0.001	0.002	-0.220	0.827
NDVI*NDVI(t-1)	-0.006	0.003	-2.241	0.030*

**Fig 3:**
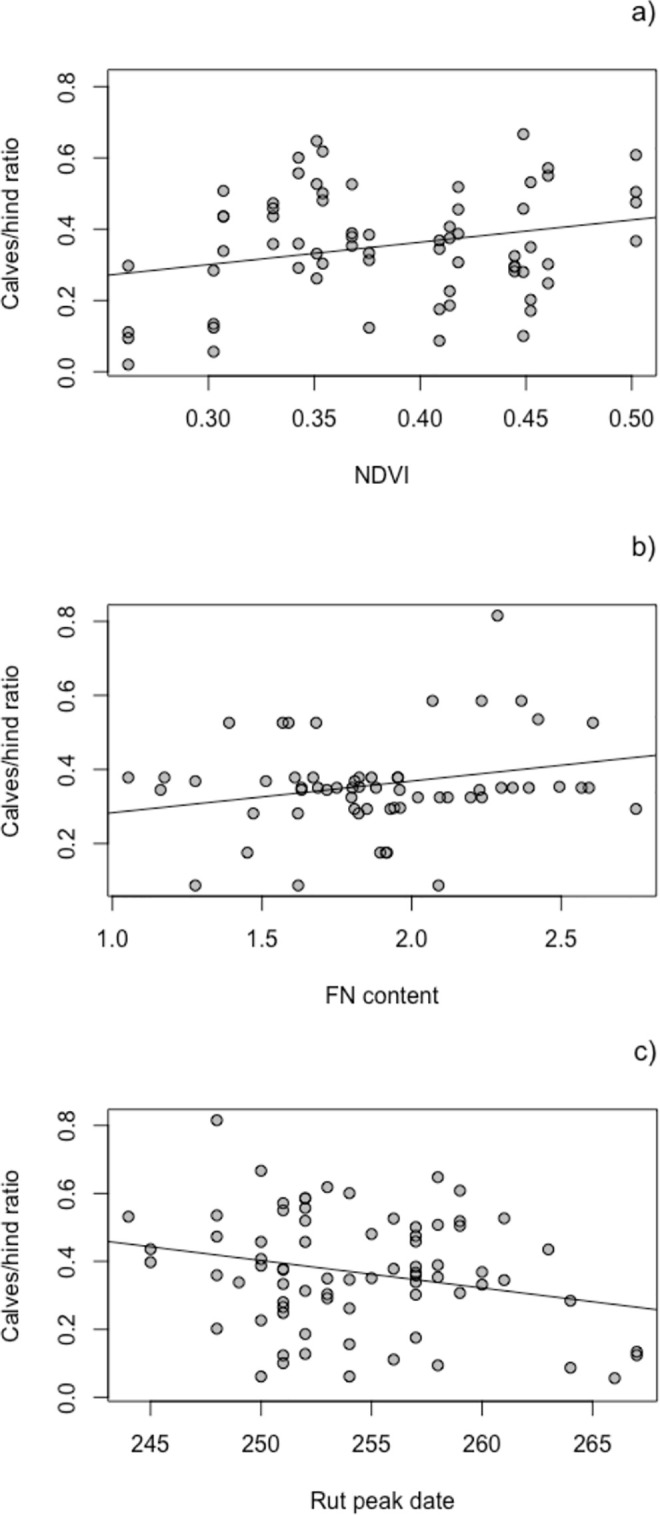
Model predictions for the effect of spring-summer NDVI (a), FN content (b) and rut peak date (c), on the percentage of calves per hind in the following year. Lines represent predictions from equations of models of Table 3, when the rest of variables were set at their mean values. Points are raw observed data.

In the models for the reproductive rate we found lower calves/hind ratio when the previous year NDVI was low (Table 3, model 3.a, Fig 3A).

**Table 3:** Models for the response variable calves/hind ratio and the fixed factors calves/hind ratio in the previous year, population density and spring-summer NDVI (3.a), FN content (3.b) or rut peak date in julian days (3.c). Observation point was added as random factor only in model b (variance = 0. 031, SD = 0.177) because of the absent of variance in the other models.

3.a)					
	Estimate	SE	t value	P	
Intercept	0.091	0.129	0.701	0.486	
NDVI	0.626	0.306	2.047	0.045	*
c/h ratio	-0.001	0.115	-0.002	0.998	
Population	0.001	0.001	0.663	0.510	
3.b)					
	Estimate	SE	t value	P	
Intercept	-1.896	0.375	-5.055	< 0.001	***
FN	0.358	0.115	3.103	0.002	**
c/h ratio	-0.349	0.349	-0.999	0.318	
Population	0.016	0.005	3.105	0.002	**
3.c)					
	Estimate	SE	t value	P	
Intercept	2.411	0.966	2.496	0.015	*
Rut peak	-0.008	0.004	-2.131	0.037	*
c/h ratio	0.127	0.121	1.056	0.295	
Population	-0.001	0.001	-0.874	0.385	

The FN content increased the calves/hind ratio observed in the next year (Table 3, model 3.b, Fig 3B), and we also found a slight positive and significant effect of the population density in the previous year on the next calves/hind ratio.

Finally, rut peak date showed a negative effect on the next calves/hind ratio (Table 3, model 3.c, Fig 3C), i. e., the later the rut occurred, the fewer females with calves were observed in the following year.

## Discussion

The results showed that a lower availability of resources affected deer body condition, delayed mating time, and decreased the reproductive rate.

We found a positive effect of the spring-summer (May-August) NDVI on the FN in September. FN content is a good indicator of nutritional status and diet quality in wild cervids such as red deer [49, 50], hence deer body condition during the rut was clearly related to prior resource availability.

Accordingly, our results showed a negative relationship between NDVI and the date of the rut peak, indicating that in environmentally favourable years (with high resource availability) the rut occurred earlier than in unfavourable years, likely due to the amount of stored resources available for reproduction [2, 5, 42]. Nevertheless, the positive effect of the NDVI of the precedent year and the significant interaction with the NDVI of the current year on the rut date, evidenced an interesting influence of the past breeding season on the current one. If in a given year the NDVI was high, most females probably reproduced, and a high investment on reproduction reduced the probability for them to breed again the next year, since females investing on gestation and lactation arrived to the following breeding season in poorer body condition than hinds who failed to breed [13, 32, 66]. Then, if the next year was drier and resources were scarce, females may not recover their nutritional status on time for reproduction, so they will breed later or even not breed. Nevertheless, when the NDVI in a given year was high, the rut seemed to occured generally early, regardless of the precedent year, likely because females had enough resources to regain energy reserves for reproduction. Therefore, to some extent the main costs of breeding seemed to occurr when females were not able to recover their nutritional condition after breeding because of the poor environmental conditions previous to the next reproductive season, so that they should respond by delaying or even suppressing ovulation in the next year.

In the Rum island population and in captive conditions in Spain, breeding red deer females arrived in worse body condition to the next mating season [32, 66]. A poor body condition has been reported to delay female oestrus in red deer [18, 67], reduce fertility or even suppress ovulation [66]. Similarly, reproductive effort affected body reserves and the subsequent ability for reproduction in bighorn ewes [61]. Hence, body reserves and reproduction appear to maintain a negative feedback: reproduction negatively affects body mass and low body mass prevents next reproduction. Our results illustrate how the environmental conditions affecting resource availability in two consecutive years can affect this interaction. In particular, our results show how good environmental conditions have opposing effects on breeding in a given year and the next one, due to the reproductive investment and the later recovery of females.

Our results on the calves/hind ratio models supported these findings, since when NDVI and FN were high, the observed number of females carrying calves in the next year was also high, indicating that higher vegetation availability allowed better body condition so that more hinds may breed. Besides, the results showed that an earlier rut, which has been related to better environmental and body conditions, was also related to higher reproductive rates, measured as the number of offspring per female observed in the following year.

Population density has been reported to increase intraspecific competition for available resources, which may affect deer body condition [62]. Nevertheless, a moderate increase in the population could increase reproduction since, according to our data (not shown), the increase in population was more related to an increase in the number of females than males, i.e, in breeding individuals. In the model for the effect of FN on the calves/hind ratio (Table 3, model 3.b) we only found a slight positive effect of population density, likely because environmental and body condition variations may affect reproduction more strongly than population density. Female age may be another population factor affecting reproduction rates [33]. However, because of the non-invasive way in which our data were collected, this variable could not be recorded.

In Mediterranean ecosystems, rainfall is a main limiting factor for forage availability [42, 68, 69]. In our study area and period, because of the increasingly dry conditions associated to global climate change predictions, resource availability may be increasingly limited, affecting hind body condition and likely that of their offspring. Nutritional stress during pregnancy and lactation has been shown to affect calf body mass and immune system [32, 70], which are related to their future survival and reproductive success [66]. Moreover, a delay in conception date might produce a mismatch between actual births and optimal calving date. If conditions are increasingly unfavourable, as predicted by the prospects of climate change for that region [68, 69], females will have more difficulties in ovulating, reproducing and lactating, and recovering body condition in the next season on time for breeding again. Therefore, according to climate change prospects, we should expect the rut and calving dates to be increasingly late over time, or even a decrease in reproduction rates, which may put at risk the future population dynamics.

The effects of resource scarcity may differ between individuals and sexes, for instance due to differences in the ability to minimize energetic costs and attain body mass and hence, their ability to reproduce [61]. In females, reproductive success is determined by their ability to yield a successful offspring, and in males, by their ability to recover body condition and develop secondary sexual characters that allow them the access to females. Thereby, if environmental conditions limit or desynchronize their potential physical condition, reproductive success in both sexes could be reduced, and even a population decline may occur, as already described in other species [71–75].

Besides, responses to current climate change depend on species and geographical locations. While some small mammals may easier adapt their reproduction to environmental changes because of their short generation time [20], big seasonal mammals such as red deer may strongly suffer the impact of global climate change. Likewise, while in some areas climate change may lead to an extended season of high-quality resource availability, in warmer and drier regions it may reduce the suitable season for breeding. In contrast to previous studies reporting an advance in red deer breeding phenology in northern Europe [13, 33, 34], this study shows how the rut timing can be delayed and female reproductive rates can decrease in response to global warming conditions. We also included a joint analysis of vegetation availability, body condition, and the effect of the previous year. These differences in the responses of the same species to global warming depending on local environmental conditions highlight how climate change is leading populations to face very different problems depending on the region. Future studies should try to reveal to what extent these responses, besides the direct effects of environmental constraints, can include adaptive adjustments that optimize their reproductive chances under the new conditions in each area.
